# Phycobiliproteins Ameliorate Gonadal Toxicity in Male Mice Treated with Cyclophosphamide

**DOI:** 10.3390/nu13082616

**Published:** 2021-07-29

**Authors:** Jorge Briseño-Bugarín, Isabel Hernández-Ochoa, Xelha Araujo-Padilla, María Angélica Mojica-Villegas, Ricardo Iván Montaño-González, Gabriela Gutiérrez-Salmeán, Germán Chamorro-Cevallos

**Affiliations:** 1 Departamento de Farmacia, Escuela Nacional de Ciencias Biológicas, Instituto Politécnico Nacional, Unidad Zacatenco, Mexico City 07738, Mexico; jorbribu@gmail.com (J.B.-B.); xelhaaraujo@gmail.com (X.A.-P.); angiemojica13@gmail.com (M.A.M.-V.); bassinpi_314@hotmail.com (R.I.M.-G.); 2 Departamento de Toxicología, Centro de Investigación y de Estudios Avanzados del Instituto Politécnico Nacional (Cinvestav-IPN), Unidad Zacatenco, Mexico City 07360, Mexico; mihernandez@cinvestav.mx; 3 Centro de Investigación en Ciencias de la Salud (CICSA), Facultad de Ciencias de la Salud, Universidad Anáhuac México, Mexico City 52786, Mexico; gabrielasalmean@yahoo.com

**Keywords:** phycobiliproteins, cyclophosphamide, testicular damage, sperm parameters

## Abstract

Cyclophosphamide (CP)—which is used to treat autoimmune diseases and cancer—is related to gonadotoxicity attributed to oxidative stress. As phycobiliproteins (PBPs) are strong antioxidants that are unexplored as protective agents against male gonadotoxicity, our work aimed to investigate the effects of PBP crude extract on testicular damage and sperm parameter alterations caused by CP in mice. Three doses of PBP (50, 100, and 200 mg/kg) were tested in the experimental groups (*n* = 8 per group), administered concomitantly with 100 mg/kg CP. After 42 days receiving PBP daily and CP weekly, body and relative testicular weights, serum testosterone levels, testicular lipoperoxidation and antioxidant enzyme activity levels, and testicular histology and sperm parameter alterations were assessed. The results showed that PBP crude extract at 200 mg/kg prevented testosterone serum reduction, body weight loss, lipoperoxidation and enzyme activity increments, and sperm parameter alterations and partially ameliorated relative testicular weight reductions and histological damage in CP-treated mice. In conclusion, we showed that PBP crude extract (200 mg/kg) mitigated oxidative damage in the testes and ameliorated alterations in sperm parameters in mice treated with CP (100 mg/kg); therefore, PBP extract could be considered as a potential protective agent against CP toxicity.

## 1. Introduction

Cyclophosphamide (CP) is a prodrug with alkylating activity that is widely used in chemotherapy, autoimmune disorders, and organ transplantation [1]. Despite its pharmacological benefits, CP has been associated with gonadotoxicity [2,3,4]. CP deteriorates sperm quality and atrophies testicular tissue in both humans and mice [5,6]. It also reduces superoxide dismutase (SOD) and glutathione peroxidase (GPX) activities in rat testes [7] and causes reductions correlated with impaired sperm quality in infertile patients [8,9]. CP metabolism generates active molecules such as phosphoramide mustard and acrolein [10], which are associated with therapeutic effect and toxicity, respectively [11]. Acrolein—a strong electrophile—creates oxidative stress conditions, modifies antioxidant enzymes [3], and reduces fertility in CP-treated patients [12]. As sperm cryopreservation—which is inaccessible to the majority of the population—is recommended before receiving chemotherapy [13,14], supplementation with phytocompounds and natural antioxidants has been proposed because they restore antioxidant balance and enhance male fertility [15]. C-phycocyanin (C-PC), allophycocyanin (APC), and phycoerythrin (PE)—known as phycobiliproteins (PBPs) [16]—are water-soluble fluorescent pigments found in cryptomonads, cyanelles, and cyanobacteria such as *Spirulina maxima* (SP) [17]. PBP extracts demonstrate antioxidant, antimicrobial, antiviral, antitumor, anti-inflammatory [18], antiteratogenic [19], antiulcerogenic [20], and nephroprotective [21] effects as well as preeclampsia-preventive activity [22]. Considering that PBPs’ activity against drug-induced gonadotoxicity is unexplored, the present work aimed to probe whether PBP extracted from SP could ameliorate testicular damage caused by multiple doses of CP in mice.

## 2. Materials and Methods

### 2.1. Reagents and Spirulina

*Spirulina maxima* powder was donated by Alimentos Esenciales para la Humanidad S.A. de C.V. (Mexico City, Mexico). Reagents and CP (CAS 6055-19-2, purity ≥ 98%) were purchased from Sigma-Aldrich Chemicals Co. (St. Louis, MO, USA) except for RANSOD and RANSEL kits obtained from Randox Laboratories (Crumlin, UK) and a testosterone enzyme-linked immunosorbent assay (ELISA) kit purchased from Cayman Chemical Co. (Ellsworth, MI, USA). All reagents were analytical grade.

### 2.2. PBP Extraction and Determination

Phycobiliproteins were extracted as described by Maurya et al. [23] by diluting SP (5 g) in phosphate-buffered saline (20 mL, 20 mM, pH 7.2). SP solution (0.25 g/mL) was frozen (−70 °C), thawed (24 ± 2 °C), shaken (5 min, 24 ± 2 °C) in darkness, and centrifuged twice at 14,000 rpm (JA-17 rotor, Beckman Coulte Co., Indianapolis, IN, USA) for 30 min at 4 °C. The supernatant was collected, PBPs were identified, and then the remaining supernatant was lyophilized and stored at −70 °C in darkness conditions. C-PC, APC, and PE optical densities (O.D.) were, respectively, determined at 615, 652, and 562 nm, while PBP concentrations were estimated as described by Bennett and Bogorad [24] and described using the following formulas:

C-PC (mg/mL) = [O.D._615_ − 0.474(O.D._652_)]/5.34, (1)

APC (mg/mL) = [O.D._652_ − 0.208(O.D._615_)]/5.09, (2)

PE (mg/mL) = [O.D._562_ − 2.41(C-PC (mg/mL)) − 0.849(APC (mg/mL))]/9.62. (3)

C-PC, APC, and PE purity indexes were estimated by dividing each O.D. by the total protein O.D. determined at 280 nm, while extraction yields were calculated from the wet basis by the following formula:

Extraction yield (mg/g) = [(PBP_C-PC, APC, or PE_ (mg/mL)) (v(mL))]/SP (g), (4)

where PBPC-PC, APC, and PE (mg/mL) represent the C-PC, APC, or PE concentration obtained, while v indicates the volume of phosphate-buffered saline.

### 2.3. Animals

The CD-1 male mice (25–30 g, 6–7 weeks old) from Bioterium of Universidad Autónoma del Estado de Hidalgo (Pachuca de Soto, Mexico) were housed in cages with access to LabDiet Rodent 5001 food (Fort Worth, TX, USA) and water ad libitum at constant temperature (24 ± 2 °C) and relative humidity (50 ± 10%) and with 12 h light/dark cycles. Conditions were maintained after and during the experimental procedures. The investigation protocol was previously approved by the Bioethical Committee of the National School of Biological Sciences of the National Polytechnic Institute (protocol code: ZOO-016-2020; 21 December 2020), and procedures with animals were performed in accordance with Mexican Official Standard NOM-062-ZOO-2001.

### 2.4. Experimental Design

**Figure 1:**
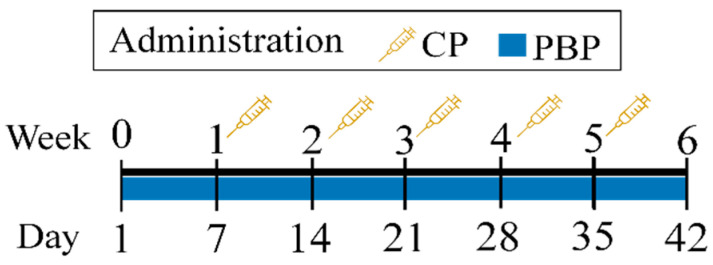
Cyclophosphamide (CP) and phycobiliprotein (PBP) extract administration. Treated male mice received daily i.g. doses of 50, 100, or 200 mg/kg PBP for 42 days concomitantly with weekly i.p. doses of 100 mg/kg CP.

Cyclophosphamide was dissolved in physiological solution (0.9% NaCl) and administered intraperitoneally at 100 mg/kg as described by Elangovan et al. [25], and Lu et al. [7], while PBPs were dissolved in phosphate-buffered saline and administered intragastrically at 50, 100, and 200 mg/kg—doubling and halving the 100 mg/kg dose reported by Castro-García et al. [22]. After an acclimatization week, treated male mice received daily i.g. doses of 50, 100, or 200 mg/kg PBP for 42 days concomitantly with weekly i.p. doses of 100 mg/kg CP (Figure 1). Control groups were included; whereby the vehicle control group received i.g. 0.9% NaCl daily for 42 days, the CP group received weekly i.g. doses of 100 mg/kg for 5 weeks, and the PBP group received daily i.p. doses of 200 mg/kg for 42 days; thus, experimental groups were defined as control, CP, PBP, PBP50 + CP, PBP100 + CP, and PBP200 + CP. At the end of treatment (day 42), the procedures described in Section 2.5, Section 2.6, Section 2.7, Section 2.8 and Section 2.9 were performed.

### 2.5. Serum Testosterone, Body Weight, and Relative Testicular Weight

Blood was collected by retro-orbital bleeding on day 42, and serum samples were separated to quantify testosterone levels using an ELISA kit. Mice were weighed and euthanized by cervical dislocation to immediately dissect the testes and epididymis and to determine the relative testicular weight.

### 2.6. Lipoperoxidation and SOD and GPX Activity in the Testes

The left testicle was homogenized in physiological solution by sonication for 40 s (output 40%, 20 kHz; Ultrasonic Homogenizer VP-050N, Taitec Corp., Koshigaya City, Saitama, Japan). Testicle homogenates were centrifuged at 10,000 rpm (1619 Rotor Universal 320 Centrifuge, Hettich^®^, Tuttlingen, DE) for 10 min at 4 °C and the supernatants were collected to evaluate lipoperoxidation and the enzymatic activity levels of SOD and GPX. Before determination, the total protein concentrations in supernatants were calculated by Bradford assay. Lipoperoxidation was determined by malondialdehyde (MDA) identification using thiobarbituric acid as proposed by Buege and Aust [26]. SOD and GPX activity levels were evaluated using RANSOD and RANSEL kits, respectively, following the manufacturers’ instructions.

### 2.7. Testicular Histology

The right testicle was conserved in buffered formaldehyde (4%, pH 7.4) until histologic observation and histomorphometry. After being dehydrated and embedded in paraffin [27], testicle sections of 5 µm thickness were prepared for hematoxylin and eosin (H&E) staining [28]. Ten stained sections per testicle were analyzed using an Olympus BX61 microscope (Olympus Corp., Shinjuku, Tokyo, Japan).

### 2.8. Histomorphometry

**Figure 2:**
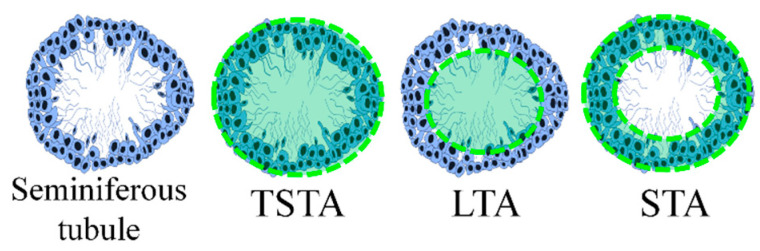
Schematic representation of the seminiferous tubule area. The total seminiferous tubule area (TSTA), lumen tubule area (LTA), and seminiferous tubule area (STA) were measured as the marked green areas indicate.

Morphology changes in seminiferous tubules were determined in five different fields from each testicle section. The total seminiferous tubule area (TSTA) and lumen tubule area (LTA) were measured with Image-Pro Plus^®^ software (Media Cybernetics Inc., Rockville, MD, USA). Next, the seminiferous tubule area (STA) was estimated as the difference between the TSTA and LTA (Figure 2).

### 2.9. Sperm Parameter Assessment

Epididymis spermatozoa were collected via flushing with M-16 medium (100 mM NaCl, 25 mM NaHCO_3_, 5.5 mM glucose, 2.6 mM KCl, 1.56 mM Na_2_HPO_4_, 0.5 mM sodium pyruvate, 1.8 mM CaCl_2_, 0.5 mM, MgCl_2_, 20 mM sodium lactate, 100 IU/mg penicillin, and 100 µg/mL streptomycin at pH 7.2 and 37 °C). The progressive motility, sperm count, and cell viability were evaluated as sperm parameters according to World Health Organization guidelines [29]. Progressive motility was determined on a glass slide, sperm were counted in a hemocytometer, and sperm viability was evaluated by eosin–nigrosin exclusion assay; a phase contrast microscope (Carl Zeiss Microscopy Co., Oberkochen, Germany) was used for all determinations.

### 2.10. Statistical Analysis

The results were analyzed by one-way ANOVA followed by the Holm–Sidak multiple comparison test. Data were represented as means ± standard error of the mean (SEM), and *p* < 0.05 was considered statistically significant. The PBP, PBP50 + CP, PBP100 + CP, and PBP200 + CP groups were compared with the control and CP groups. Analysis was performed and graphs were produced in SigmaPlot v12.0 software. All samples were evaluated in triplicate.

## 3. Results

### 3.1. PBP Determination

The PBP concentrations, purity index values, and extraction yields are indicated in Table 1. C-PC, APC, and PE, respectively, corresponded to 69.04, 30.34, and 0.62% of the PBPs extracted from SP, with C-PC being 2.3- and 111.5-fold more abundant than APC and PE, respectively. Additionally, the purity index and extraction yield of C-PC were higher than the purity index values and extraction yields of APC and PE.

### 3.2. Serum Testosterone, Body Weight, and Relative Testicular Weight

**Figure 3:**
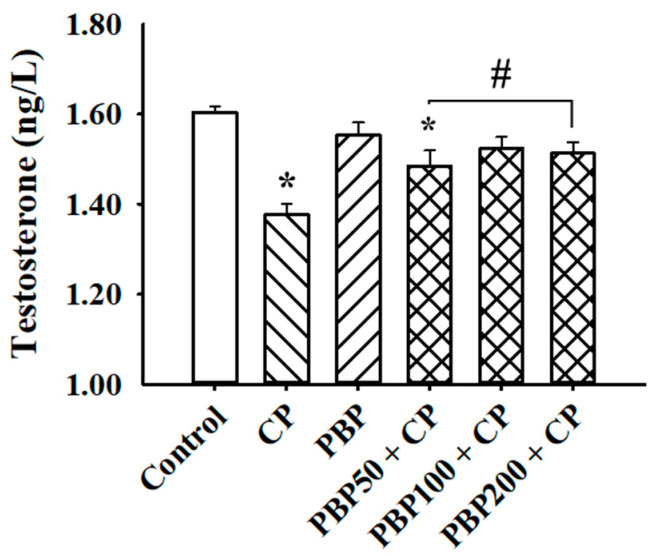
Effects of PBPs on testosterone levels of CP-treated mice. Testosterone levels evaluated in serum after treatment ended are represented as means ± SEM. Results analyzed by one-way ANOVA followed by Holm–Sidak test. * Indicates *p* < 0.05 vs. control group; # indicates *p* < 0.05 vs. CP group.

**Figure 4:**
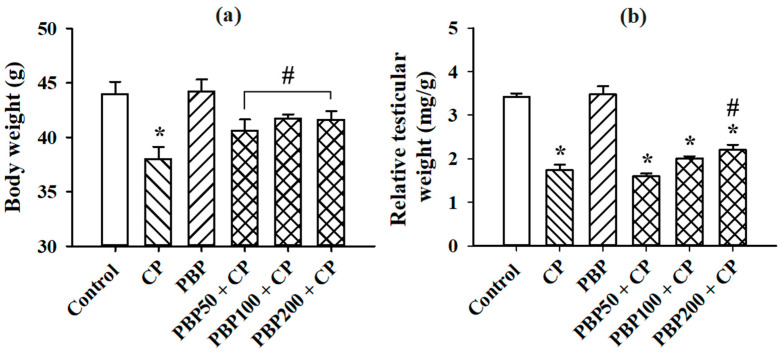
Effects of PBPs on body weight and relative testicular weight of CP-treated mice: (**a**) body weights measured at end of treatment, (**b**) relative testicular weights. Means ± SEM analyzed by one-way ANOVA followed by Holm–Sidak test. * Indicates *p* < 0.05 vs. control group; # indicates *p* < 0.05 vs. CP group.

Figure 3 shows the results obtained from the testosterone serum determination. PBP at 100 and 200 mg/kg prevented the reductions in testosterone levels observed in the CP group. Although the PBP50 + CP group showed testosterone levels statistically lower than control, PBP50 + CP testosterone levels were 7.9% higher than the CP group. The body weight results (Figure 4a) showed that PBP at 50, 100, and 200 mg/kg prevented weight loss caused by CP, while the relative testicular weight data (Figure 4b) indicated that all groups receiving CP decreased their relative testicular weight compared to control; however, the statistical analysis showed that the PBP200 + CP group presented relative testicular weights higher than those of the CP group, suggesting that the PBP 200 mg/kg dose ameliorated testicular weight reduction.

### 3.3. Lipoperoxidation and SOD and GPX Activity in the Testes

**Figure 5:**
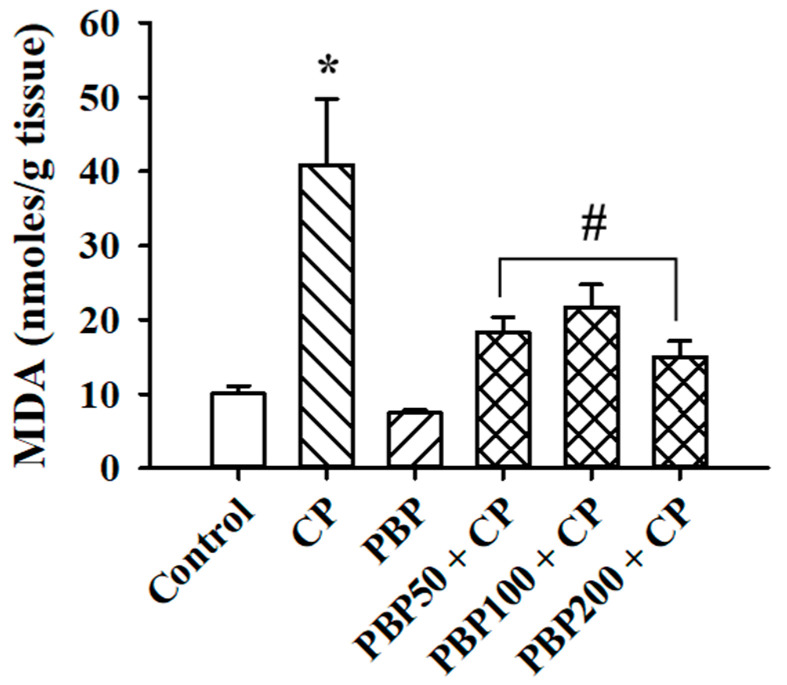
Effects of PBPs on testis lipoperoxidation of CP-treated mice. Malonaldehyde (MDA) was measured as an indicator of lipoperoxidation. Means ± SEM analyzed by one-way ANOVA followed by Holm–Sidak test. * Indicates *p* < 0.05 vs. control group; # indicates *p* < 0.05 vs. CP group.

**Figure 6:**
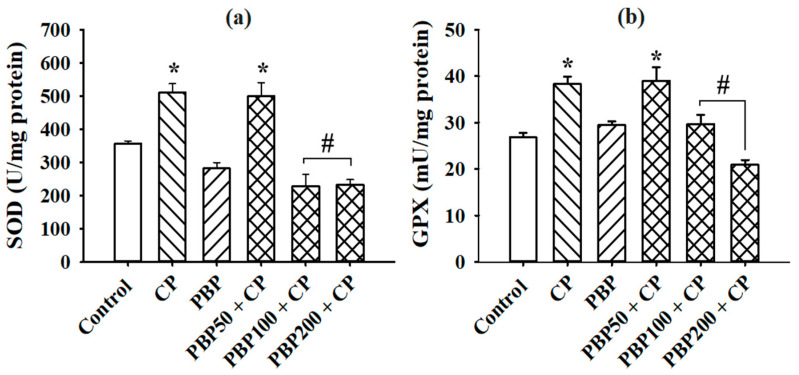
Effects of PBPs on SOD and GPX enzyme activities of CP-treated mice: (**a**) superoxide dismutase (SOD), (**b**) glutathione peroxidase (GPX). The activity levels were evaluated in mice testicles at the end of treatment. Means ± SEM analyzed by one-way ANOVA followed by Holm–Sidak test. * Indicates *p* < 0.05 vs. control group; # indicates *p* < 0.05 vs. CP group.

The MDA levels, determined as lipoperoxidation indicators, are shown in Figure 5. MDA in the CP group was fourfold greater than the control, while PBP50 + CP, PBP100 + CP, and PBP200 + CP groups maintained MDA at similar levels to the control group, suggesting a protective effect of PBP (50–200 mg/kg) against testicular lipoperoxidation induced by CP. On the other hand, as presented in Figure 6, the enzymatic activity of SOD and GPX increased in the CP and PBP50 + CP groups compared with the control, while the PBP100 + CP and PBP200 + CP groups showed similar SOD and GPX activity levels to the control group.

### 3.4. Testicular Histology

**Figure 7:**
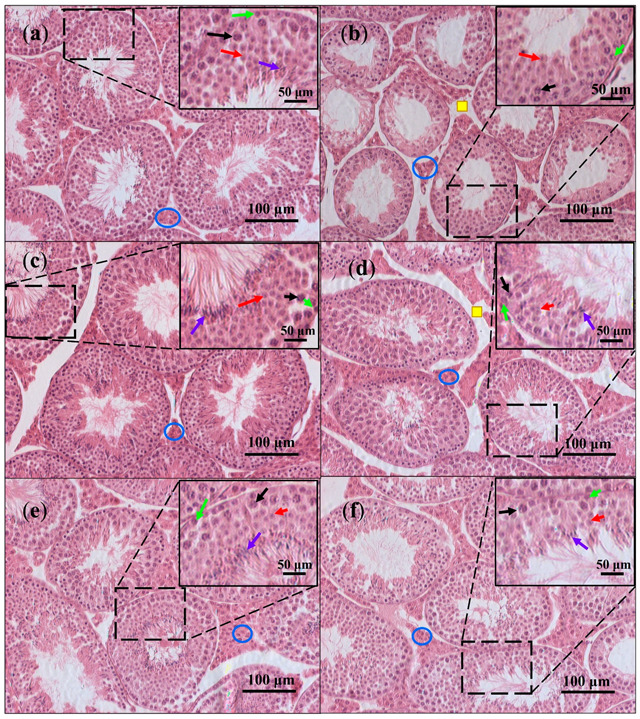
Effects of PBPs on the testicular histology of CP-treated mice: (**a**) The control group presented spermatogonium (green arrows), spermatocytes (black arrows), spermatids (red arrows), and mature spermatids (purple arrows) in seminiferous tubules and Leydig cells in interstitial space (blue circles); (**b**) the CP group showed small seminiferous tubules, large interstitial spaces (yellow squares), and several spermatids; (**c**) PBP, (**d**) PBP50 + CP, (**e**) PBP100 + CP, and (**f**) PBP200 + CP groups presented preserved germinal cells; however, large interstitial spaces were identified in PBP50 + CP (**d**). Boxes in the upper right corner are maximizations (X2) from specific field areas, as indicated by dashed lines. H&E-stained sections observed at 10×.

Figure 7 shows representative micrographs from histological observations. The control and PBP groups presented intact seminiferous tubules where spermatogonium, spermatocytes, and spermatids were identified. Similarly, the PBP50 + CP, PBP100 + CP, and PBP200 + CP groups preserved germinal cells from the basal membrane to the tubule lumen, while the CP group showed small tubules and large spaces in both the tubule lumen and interstitium, indicating possible depletion of cells, especially mature spermatids and Leydig cells. As interstitial spaces were also observed in PBP50 + CP micrographs, the testicular histology indicated that PBPs at the 50 mg/kg dose partially ameliorated damage caused by CP, while the 100 and 200 mg/kg doses improved testicle histology.

### 3.5. Histomorphometry

The TSTA, LTA, and STA are shown in Table 2. Although large spaces in the seminal lumen were observed only in the CP group micrograph (Figure 7b), the LTA diminished in all the groups compared with the control, while TSTA was reduced only in the CP group. STA, which represented the seminal tubule cellular structure (Figure 2), was decreased in the CP group compared with the control, confirming tubule reduction in the CP micrograph (Figure 7b). The PBP50 + CP, PBP100 + CP, and PBP200 + CP groups presented STA values lower than the control but greater than the CP group, indicating partial improvement of testicular CP damage. Interestingly, TSTA and STA levels in the PBP group (without CP) were 12.8 and 21.9% higher than the control, respectively, suggesting that PBP consumption (200 mg/kg) contributed to germinal cell increase.

### 3.6. Sperm Parameters

**Figure 8:**
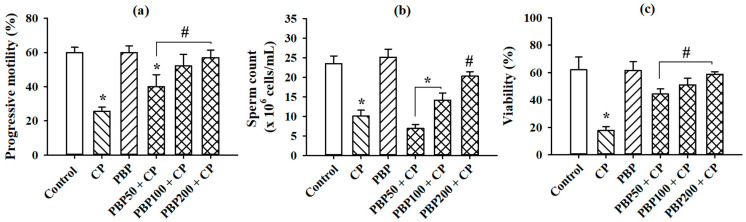
Effects of PBPs on sperm parameters of CP-treated mice: (**a**) progressive motility; (**b**) sperm count; (**c**) cell viability. Means ± SEM analyzed by one-way ANOVA followed by Holm–Sidak test. * Indicates *p* < 0.05 vs. control group; **#** indicates *p* < 0.05 vs. CP group.

The progressive motility, sperm count, and sperm viability—evaluated as parameters of sperm quality—are shown in Figure 8. The three evaluated parameters were reduced in the CP group compared with the control. PBP50 + CP, PBP100 + CP, and PBP200 + CP maintained cell viability; PBP100 + CP and PBP200 + CP preserved progressive motility; and PBP200 + CP sustained sperm count at similar levels to the control. The data suggested that PBPs at 200 mg/kg were required to recover sperm parameter alterations caused by CP administration in mice.

## 4. Discussion

Although CP is used to treat autoimmune diseases and cancer, reproductive organ toxicity has been shown in patients and experimental models [30,31,32]. As CP toxicity is attributed to oxidative stress [10], and PBPs demonstrate strong antioxidant properties [33], we explored the effects of PBP crude extract on the testicular damage caused by multiple CP doses in mice. In PBP crude extract obtained from SP, we identified a higher C-PC percentage (69.04%) than APC (30.34%) and PE (0.62%) (Table 1). C-PC, which is related to high antioxidant capacity [16], was reported in PBP crude extracts from SP by Rodríguez-Sánchez et al. at a low percentage (C-CP, 47%) [21] and by Walter et al. at a reduced concentration (C-PC, 0.237 mg/mL) [34] as compared with our results; however, the C-PC purity index reported by Walter (0.8) correlates with our C-PC purity index result (0.7), with both index values considered to be of food grade (purity index ≥ 0.7) [35]. PBP concentrations (50, 100, and 200 mg/mL) were selected according to the results observed by Castro-García et al. [22] in a rat preeclampsia model, while the duration of CP treatment was determined considering the spermatogenesis cycle [36].

Our results are in agreement with the CP effects previously reported by Elangovan et al. [25], Lu et al. [7], and Iqubal et al. [37] including reductions in serum testosterone, body weight, relative testis weight, and sperm parameter alterations; increases in lipoperoxidation and enzyme activity (SOD and GPX); and testicle damage observed by histology. The PBP crude extract mitigated certain CP effects, depending on the PBP dose administered. In particular, PBP at 200 mg/kg prevented changes in testosterone serum, body weight, lipoperoxidation, enzyme activity, and evaluated sperm parameters while partially ameliorating relative testicular weight loss and histological damage. Body weight loss is attributed to appetite disruption caused by sensory taste cell modifications after CP administration in mice [38]. Protein consumption is recommended to avoid weight loss and malnutrition in patients receiving chemotherapy [39,40]; thus, body weight recuperation observed in CP-treated mice at PBP doses of 50, 100, and 200 mg/kg was probably an effect of the protein content in the PBP crude extract.

The antioxidant effects of PBPs have been previously demonstrated [17,41], which were confirmed in our results, whereby lipoperoxidation and SOD and GPX enzymatic activity increments were prevented with PBP doses of 100 and 200 mg/kg. As SOD and GPX activity, as well as lipoperoxidation [42], are proportional to oxidative stress conditions [43,44,45] and because the antioxidant effects of PBP are related to the capacity of C-PC to trap reactive molecules such as acrolein [46], oxidative stress—generated as a consequence of acrolein interactions—was possibly reduced by the presence of C-PC in the PBP crude extract; however, the PBP crude extract at 200 mg/kg—the highest concentration tested in our study—partially ameliorated the relative testis weight reduction and testicular damage observed by histology. Decrements in relative testicular weight were probably related to STA reductions and decreases in Leydig cells and spermatids in the CP group. Despite our histological results revealing that spermatogonia were preserved after multiple CP doses as previously described by Drumond et al. [47], the interstitial spaces observed in the CP and PBP50 + CP groups suggest Leydig cells loss. Gu et al. [48] related this to the autophagy process of Leydig cells that is induced by CP. As Leydig cells maintain testosterone secretion [49], observed testosterone reductions confirm Leydig cell loss. Although testosterone levels and interstitial spaces improved in PBP 100 and 200 mg/kg groups, suggesting that Leydig cells were recovered, STA values and relative testicular weights were lower than the control, suggesting that cell populations were deteriorated. Interestingly, the PBP (200 mg/kg)-treated group without CP presented higher STA values than the control, probably because the antioxidant activity of PBPs reduced the oxidant molecules required for regular apoptosis processes [50].

On the other hand, sperm parameters such as progressive motility, sperm count, and cell viability were completely recovered at the PBP dose of 200 mg/kg. The effects of the PBP crude extract on sperm parameter alterations in CP-treated mice are attributable to the antioxidant activities of PBPs because damage observed in sperm is directly related to oxidative stress conditions. For example, decreased sperm motility is a result of flagellum defects caused by oxidative damage in the tubulin [51], reduced sperm count is related to spermatogenesis inhibition or apoptosis induction by oxidant molecules, and cell viability is associated with apoptosis triggered by oxidative stress [52].

The NADPH oxidase system (NOX)—embedded in the plasma membrane—catalyzes O2^•−^ production through the oxidation of NADPH. NADPH oxidase isoform 5 (NOX5) is a potential candidate for the ROS-generating system in spermatozoa [53,54]. NOX5 can be directly activated by PKCα independent of an increase in intracellular calcium [55]. Acrolein, which is likely the agent responsible for the toxicity of CP to spermatozoa, is known to activate PKCα [56]; hence, overactivation of NOX5 may be largely responsible for the pro-oxidative impact of CP on spermatozoa. Intracellular free bilirubin generated by heme oxygenase is known to inhibit various isoforms of NADPH oxidase in low nanomolar concentrations [57], although its specific effect on the NOX5 form has not been reported. Phycocyanobilin—a compound present in the chromophores of phycocyanin and allophycocyanin [17]—is converted within cells to phycocyanorubin, which is nearly identical in structure to bilirubin [58]; this may explain why phycocyanobilin has been found to mimic the NADPH oxidase-inhibitory effect of biliverdin or bilirubin in cell cultures. Our results, therefore, suggest that bilirubin and phycocyanobilin can function as inhibitors of NOX5, thereby protecting spermatozoa from acrolein. This possibility could be easily tested in vitro.

As PBP crude extract was studied in the present work, the beneficial effects observed against CP gonadotoxicity in male mice could be increased by other phytochemicals with antioxidant properties such as the polyphenols [59] and polysaccharides phycobiliproteins (PBPs) [60] previously identified in SP. Although our results suggest that PBPs ameliorate CP damage, this study was limited to evaluating healthy mice; hence, the effects of PBPs must be assessed in an animal model of cancer or autoimmune disease to exclude an inactivation of the therapeutic effects of CP.

## 5. Conclusions

Phycobiliproteins crude extract at 200 mg/kg ameliorated gonadotoxicity caused by CP in male mice. Alterations in testosterone serum, body weight, lipoperoxidation, enzyme activity, and sperm parameters were completely prevented, while relative testicular weight loss and histological damage were attenuated; however, additional CP function tests are recommended.

## Figures and Tables

**Table 1:** PBP concentrations, purity index values, and extraction yields.

PBP	Concentration (mg/mL)	Purity Index	Extraction Yield (mg/g, wb SP)
C-PC	2.23 ± 0.02	0.7 ± 0.02	8.93 ± 0.44
APC	0.98 ± 0.14	0.3 ± 0.02	3.91 ± 0.56
PE	0.02 ± 0.001	0.005 ± 0.001	0.08 ± 0.004

Mean ± standard deviation (SD) from triplicates; C-PC: phycocyanin; APC: allophycocyanin; PE: phycoerythrin; PBPs: phycobiliproteins; SP: *Spirulina maxima*; wb: wet basis.

**Table 2:** Effects of PBP on seminiferous tubule areas of CP-treated mice. The total seminiferous tubule area (TSTA), lumen tubule area (LTA), and seminiferous tubule area (STA) were determined.

Group	TSTA (µm^2^)	LTA (µm^2^)	STA (µm^2^)
Control	50,082 ± 945.4	10,771 ± 342.4	39,311 ± 755.1
CP	24,764 ± 511.3 ^a^	7763 ± 251.0 ^a^	17,001 ± 396.9 ^a^
PBP	56,481 ± 1535 ^ab^	8575 ± 600.2 ^a^	47,906 ± 1249 ^ab^
PBP50 + CP	35,576 ± 1247 ^b^	7452 ± 600.4 ^a^	28,124 ± 996.4 ^ab^
PBP100 + CP	38,683 ± 1237 ^b^	8209 ± 407.6 ^a^	30,474 ± 1085 ^ab^
PBP200 + CP	40,320 ± 1898 ^b^	9217 ± 659.9 ^a^	31,102 ± 1855 ^ab^

Means ± SEM analyzed by one-way ANOVA followed by Holm–Sidak test; ^a^ *p* < 0.05 vs. control group; ^b^ *p* < 0.05 vs. CP group.
